# Prevalence and Risk Factors for Chronic Edema in U.K. Community Nursing Services

**DOI:** 10.1089/lrb.2018.0086

**Published:** 2019-04-22

**Authors:** Christine J. Moffatt, Rebecca Gaskin, Martina Sykorova, Eleanor Dring, Aimee Aubeeluck, Peter J. Franks, Paul Windrum, Gregoire Mercier, Lorraine Pinnington, Isabelle Quéré

**Affiliations:** ^1^ School of Social Sciences, Nottingham Trent University, Nottingham, United Kingdom.; ^2^ Montpellier Medecine Vasculaire, EA2992, Universite Montpellier I, CHU Saint Eloi, Montpellier, France.; ^3^ Copenhagen Wound Healing and Lymphoedema Centre, Bisperberg University Hospital, Copenhagen, Denmark.; ^4^ School of Health Sciences, University of Nottingham, Royal Derby Hospital Centre, Derby, United Kingdom.; ^5^ Nottingham University Business School, University of Nottingham, Jubilee Campus, Nottingham, United Kingdom.; ^6^ School of Health Sciences, University of Nottingham, Queens Medical Centre, Nottingham, United Kingdom.; ^7^ Centre for Research & Implementation of Clinical Practice, London, United Kingdom.; ^8^ School of Medicine, University of Nottingham, Royal Derby Hospital Centre, Derby, United Kingdom.

**Keywords:** chronic edema, leg ulcer, cellulitis, community nursing services, prevalence, risk factors, lymphedema, lymphoedema

## Abstract

***Background and Study Design:*** Chronic edema (CO) is believed to be a major clinical problem within community nursing services in the United Kingdom. This study was undertaken as part of the LIMPRINT international study to determine the number of people with CO and its impact on health services.

***Methods and Results:*** Three urban-based community nursing services participated in the United Kingdom with prospective evaluation for 4 weeks of all patients receiving nursing care using a questionnaire-based interview and clinical assessment using the LIMPRINT tools. Of the total 2541 patients assessed, 1440 (56.7%) were considered to have CO, comprising Leicester City [768/1298 (59.2%)], Nottingham West [124/181 (68.5%)], and Nottingham City [548/1062 (51.6%)]. The mean age for women with CO was 78.6 (standard deviation [SD] 12.8) years and that for men with CO was 72.9 (SD 14.5). More patients with CO suffered from diabetes (32.1% vs. 27.9%, *p* = 0.027), heart failure/ischemic heart disease (27.3% vs. 14.0%, *p* < 0.001), and peripheral arterial occlusive disease (5.5% vs. 1.9%, *p* < 0.001). By far the greatest association was with the presence of a wound (73.6% vs. 37.9%, *p* < 0.001). Cellulitis affected 628 patients (24.7%) and 688 patients (47.8%) had a concurrent leg ulcer. Rates of reduced mobility (71.6% vs. 61.9%) and obesity were higher in those with CO. Six independent factors associated with CO were service location, age, ethnicity, obesity, heart failure, and the presence of a wound.

***Conclusion:*** CO is a major and growing health care problem within primary care that has been previously unrecognized and requires effective service provision.

## Introduction

### Chronic edema

Chronic edema (CO) is a major clinical problem worldwide that has many important secondary consequences. The term “chronic edema” is now commonly used in place of “lymphedema” as this encompasses all forms of edema that persist for 3 months, irrespective of the etiology and corresponding comorbidities and risk factors.^1^ CO is associated with many long-term conditions such as cancer and diabetes and is also related to reduced mobility and obesity, both of which are expected to escalate exponentially in Western populations for the next 10–15 years.^2,3^ Although CO has potentially life-threatening consequences, the prevalence and impact of the problem remain poorly understood, particularly in community care settings.^4,5^

To date, the focus of previous research has been to estimate prevalence in specific patient groups;^6^ however, since CO is the final common pathway for many conditions, it is important that prevalence is examined among heterogeneous populations. One earlier study of a mixed London-based population estimated the prevalence of CO to be 1.33 per 1000^1^ and more recently a higher level of 3.93 per 1000 in an urban population in the East Midlands (United Kingdom).^4^

### The context of community nursing in the United Kingdom

Government policy in the United Kingdom over recent decades has focused on the shift of care from acute hospital services to primary care.^7^ These changes have major implications for community nursing due to demographic changes of an aging population. It is estimated that by 2039, the U.K.'s population will include 3.5 million people aged 85 years and over, with many over the age of 100 years, which mirrors other Western countries.^8^ The rise in the number of elderly patients and improved life expectancy are linked to an increase in the presence of chronic polymorbidity. It is estimated that by 2018, nearly 3 million people will suffer from three or more coexisting long-term conditions.^9^ These changes indicate that the demand for community nursing will increase over the coming decades.

A review of community nursing by the Queens Nursing Institute in 2014 highlighted the current challenges in the United Kingdom.^10^ They reported that many nurses work in silos with little access to professional support. Services were unable to be responsive to sudden changes in demand due to budgetary restrictions and lack of capacity within existing teams to extend caseloads when required.

The review also highlighted inconsistent workloads and inappropriate use of staff skill mix as major factors influencing the delivery of care. The extent and level of information technology available to community nursing services were found to be grossly inadequate, with limited support for implementation and training. The high level of documentation involved in nursing care created additional pressures for staff and reduced the time available for patient care.

The recent publication of the framework for nursing, midwifery, and care staff^11^ has highlighted the need for a more integrated health and social care service.

### CO and community nursing

Community nurses have been the main provider of care for leg ulceration and pressure ulcers for many decades and absorb a large proportion of health resources.^12^ Reduction in the incidence of pressure ulceration has been the subject of government targets with a more recent focus on leg ulceration and wound assessment (CQUIN program).^13^

CO has not been recognized as part of a community nursing remit. The reasons behind this are complex. There is lack of professional recognition that CO occurs concurrently with venous ulceration and other wounds.^1^ In addition, these complex patients have multiple comorbidities and reduced mobility placing them at greater risk of CO. However, there is limited evidence of the size and scope of the problem and more generally a lack of accurate information about the current patient profile within community nursing services in the United Kingdom.

A recent wound prevalence study undertaken in two areas of Nottingham in the United Kingdom showed the prevalence of CO was low (9%). The reasons for this included a lack of community nursing knowledge and skills to recognize the problem and the lack of inclusion in nursing documentation.^14^ These underpinning factors informed this study design that had the following hypotheses:

- Patients with CO would have a high prevalence within community nursing services
- Patients with leg ulceration would have concurrent CO
- CO would be associated with reduced mobility and obesity
- Patients with CO would have greater comorbidities than those without
- Cellulitis would be associated with CO.

This study formed part of an international epidemiology study LIMPRINT to define the prevalence and impact of CO in health services in different countries and health care systems. The development and validation of the methods for the main study are reported separately.^15^

To understand the current scale of the problem within community nursing services, the study was designed with two related aims:

- To estimate the prevalence and impact of CO among a heterogeneous population within the community nursing services of three urban geographical areas in the East Midlands (United Kingdom) and to determine the proportion that had concurrent leg ulceration.
- To identify risk factors associated with CO.

## Materials and Methods

### Setting and sampling frame

This cross-sectional study was carried out in three city locations, Leicester City (population 337,653**)**, Nottingham City (population 325,000), and Nottingham West (population 112,000). Data were obtained using the same methodology in each area.

### Identification of patients through the creation of master lists

A master list of all patients in receipt of community nursing care was generated from the National Health Service (NHS) systems. The list was divided into the nursing teams responsible for their care within each locality. Nursing team leaders allocated a review of all patients for 4 weeks. Patient information was provided to all patients by the responsible community nurse 3 days before the clinical assessment and consent was obtained from the patient on the day the screening was undertaken. Clinical review occurred in the normal setting the patient was seen, this included clinic rooms and the patient's own home. The reasons for all exclusion were recorded. All forms were checked against the master list to ensure complete data capture.

### Screening for CO

People with CO were identified initially by the use of a standard assessment using the “Pitting edema Test” that measures the site and depth of swelling in an edematous area when pressure is applied by the thumb. A positive result is indicated if a “pit” remains after removal of pressure. This is a well-established clinical technique, but inter-rater reliability was further assessed for its use in the LIMPRINT study. Levels of agreement were found to be high between general nurses performing the technique and a clinical expert (gold standard) in Japan.^16^ Edema was judged to be chronic if it had been present for 3 months or more. Participants were also selected on the basis of the following criteria:

- Adults (>18 years) of both genders
- All ethnic groups
- Receiving community nursing care.

### Core data set

A modified core data set was used based on the LIMPRINT study.^15^ A detailed classification of primary and secondary causes of CO was excluded as this lay beyond the skill set of community nurses. Details of ethnicity, smoking status, and nursing care requirements were added.

The following data were collected using a standardized questionnaire:

- social demographics and care: age, gender, ethnicity, patient's general practitioner, reason for receiving community nursing care, frequency of nursing visits;
- presence and site of CO using a body map, details and duration of CO, cellulitis history, leg ulceration and wounds, pitting test, stemmer sign, and soft/hard tissues;
- lower limb mobility status (bedbound, chair-bound, walks with walking aid, walks unaided);
- upper body mobility (full range of movement, limited range of movement, and normal function);
- obesity status (underweight, normal weight, obese, and morbidly obese);
- relevant comorbidities: diabetes mellitus, neurological disorders, heart failure/ischemic heart disease, peripheral arterial occlusive disease, and smoking status.

The questionnaire was piloted with five nurses and all community nurses were trained and assessed as competent by the research team. An inter-rater reliability study was undertaken to assess community nursing accuracy in detecting CO after training compared with a gold standard of assessment (Lymphology expert). This was undertaken with 19 patients randomly selected from the main cohort (10 with CO defined by the community nurse and 9 without). Kappa coefficients (>0.85) indicated community nurses were likely to under predict the presence of CO compared with the expert assessor. However, there were no false negatives. This indicates that the rates of CO may be slightly lower than the true prevalence.

### Data collection procedures and approvals

Staff in each community nursing team screened their total caseloads for consent and inclusion irrespective of the reason they were receiving nursing care. A unique patient identification code was issued to avoid “double counting” for all cases. Master identifier lists were retained by each service manager to ensure that anonymity was maintained and for data protection purposes. Tissue viability specialist services and managers undertook quality checks and a central project manager checked data completeness. Approval for the project was granted by the Research and Innovation Department of Leicester and Nottingham, and the trust data protection and senior management teams in each area.

### Data analysis

Data were entered onto an Excel spreadsheet and exported into Stata 11 where statistical analyses were undertaken. The analysis was undertaken by examining the differences between the different city locations. This included comparison of crude prevalence rates in the patients receiving community nursing care and the ethnic profiles of each location. The combined data set was used to examine the associations between age and gender with the presence of CO. Logistic regression analysis was undertaken to examine the relationship between the presence of CO and comorbidities including obesity and poor mobility. Finally, an analysis was undertaken to identify independent factors that were associated with the presence of CO in the cohort.

## Results

**FIG. 1:**
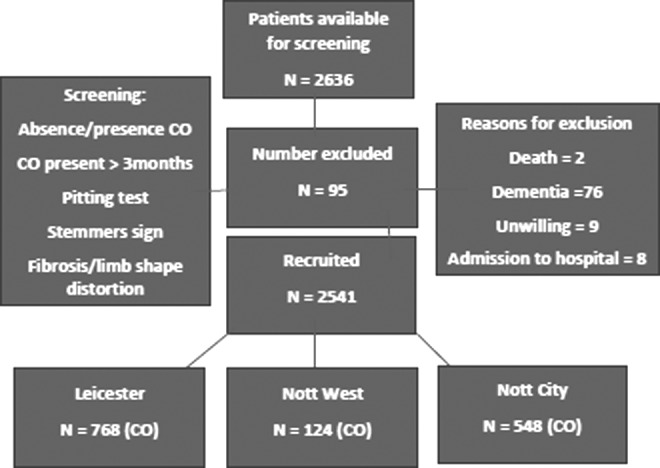
Study flow of patient screening and entry.

**FIG. 2.**
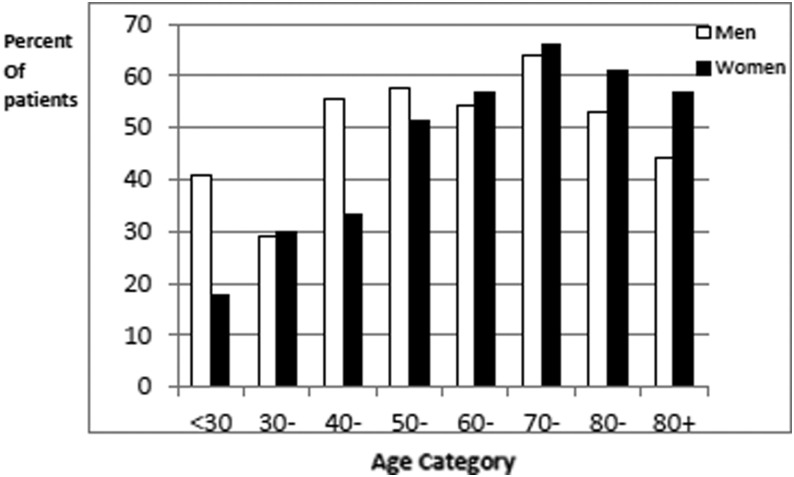
Percentage of community patients with chronic edema.

A total of 2636 patients were receiving community nursing care in the three cities during the study period. Of these 2541 (96.4%) patients consented for inclusion (Fig. 1). Ninety-five patients were excluded for the following reasons: death (2), admission to hospital (8), dementia (76), and unwilling to participate (9). The total number suffering with CO in the three cities was 1440 of 2541 (56.7%). In Leicester City, 768 of 1298 (59.2%) presented with CO compared with 124 of 181 (68.5%) in Nottingham West and 548 of 1062 (51.6%) in Nottingham City. Key demographic and clinical data are presented in Table 1. The mean age for women with CO was 78.6 (standard deviation [SD] = 12.8) years and that for men with CO was 72.9 (SD = 14.5), which was similar to the overall population of patients seen by community services (77.1 and 72.8 years, respectively) (Figure 2). The population with CO were predominantly white Caucasian but with a proportion of South Asian descent.

**Table 1.** Total Group Analysis Chronic Edema and Ethnicity in Three City Areas in the United Kingdom

*Distribution of CO in patients treated in the community services*	*Nottingham City*		*Nottingham West*		*Leicester*		*Total*	
	N	*%*	N	*%*	N	*%*	N	*%*
Chronic edema	548	51.6	124	68.5	768	59.2	1440	56.7
No edema	514	48.4	57	31.5	530	40.8	1101	43.3
Total	1062		181		1298		2541	

	*Nottingham City*		*Nottingham West*		*Leicester*		
*Ethnicity of all participants*	N	*%*	N	*%*	N	*%*	*Total*
White British/Irish/other White	948	89.6	179	100	982	75.8	2109
Asian/Asian British	36	3.4	0	0	251	19.4	287
Black African/Caribbean/Black British	52	4.9	0	0	36	2.8	88
Other ethnic group	8	0.8	0	0	14	1.1	22
Mixed/multiple ethnic background	14	1.3	0	0	13	1.0	27
Total	1058		179		1296		2533

CO, chronic edema.

Comorbidities were common with more patients with CO also suffering from diabetes (32.1% vs. 27.9%, *p* = 0.027) and heart failure/ischemic heart disease (27.3% vs. 14.0%, *p* < 0.001), Table 2. Peripheral arterial occlusive disease was more frequent in the CO group (5.5% vs. 1.9%, *p* < 0.001). By far the greatest association was with the presence of a wound (73.6% vs. 37.9%, *p* < 0.001).

**Table 2.** Comorbidities

*Comorbidities*	*No edema*		*Chronic edema*		*OR (95% CI)*	p
	N	*%*	N	*%*		
Diabetes						
Absent	794	72.1	978	67.9		
Present	307	27.9	462	32.1	1.22 (1.03–1.45)	0.022
Heart failure/CHD						
Absent	947	86.0	1047	72.7		
Present	154	14.0	393	27.3	2.31 (1.88–2.84)	<0.001
Neurological disease						
Absent	953	86.6	1258	87.4		
Present	148	13.4	182	12.6	0.93 (0.74–1.18)	0.551
Peripheral arterial occlusive disease						
Absent	1080	98.1	1361	94.5		
Present	21	1.9	79	5.5	2.98 (1.83–4.86)	<0.001
Smoking						
No	470	91.4	481	87.8		
Yes	44	8.6	67	12.2	1.49 (0.99–2.22)	0.051
Wound present						
Absent	682	62.1	380	26.4	1.00	
Present	417	37.9	1059	73.6	4.56 (3.85–5.40)	<0.001

CHD, chronic heart failure; CI, confidence interval; OR, odds ratio.

A history of cellulitis was reported as a common complication affecting 628 patients (24.7%), of whom 411 (16.2%) were reported in the last year (Table 3). Although 224 (9%) reported only one episode, 11 had suffered >5. Of the total experiencing cellulitis, 49 patients had required hospitalization for intravenous antibiotics during the last year and a further 402 patients (16.2%) were receiving repeat prescriptions for antibiotics. Wounds occurred concurrently with CO in 73.6% of cases. Of the total with CO, 28.4% could walk unaided compared with 38% in those without CO. Obesity and morbid obesity occurred in 34.9% of those with CO compared with 13.5% in those unaffected.

**Table 3.** Cellulitis in Patients with Chronic Edema

*Cellulitis history*	N	*Total*	*%*
Ever had cellulitis			
Yes	628	2534	24.7
Infection in past year	411	2123	16.2
Number of infections			
0	2069		83.5
1	224		9.04
2	88		3.55
3	49		1.98
4	26		1.05
5	10		0.40
>5	11		0.40
Hospitalization			
0	2429		97.94
1	38		1.53
2	11		0.44
3–4	2		0.08
Repeat antibiotics	402	2468	16.29

Less than a third (29.2%) were receiving nursing care once a week with >40% requiring twice weekly visits and 13.7% requiring daily visits (Table 4). A further 32 (2.4%) patients required twice or three times daily visits including the night service. Most patients were receiving care at home (83%) with a small proportion (17%) attending a clinic for treatment. These results are somewhat different from those of the patients with no edema, who were seen more frequently than the patients with edema. This is probably due to the serious underlying conditions that required care.

**Table 4.** Frequency and Site of Provision of Community Nursing Care

*Community nursing care*	*No edema*		*Chronic edema*		*OR (95% CI)*	p
	N	*%*	N	*%*		
Place						
Clinic	98	8.9	244	17.0	1.00	
Home	1003	91.1	1194	83.0	0.48 (0.37–0.61)	<0.001
Visits per week						
Once per week	310	32.6	398	29.2	1.00	
Twice per week	251	26.4	560	41.2	1.74 (1.41–2.14)	
More than 2/week	124	13.0	184	13.5	1.16 (0.88–1.52)	<0.001
Once per day	238	25.0	187	13.7	0.61 (0.48–0.78)	
More than once per day	29	3.1	32	2.4	0.86 (0.51–1.45)	

Univariate analysis identified a number of factors associated with an increased risk of having CO, these are presented in Table 5. Diabetes mellitus, heart failure, peripheral arterial occlusive disease, the presence of a wound or leg ulcer, and frequency of treatment were all identified. These factors were entered in a multivariate model that confirmed the following six independent risk factors: city location of the service, increased age, ethnicity (white Caucasian), obesity and morbid obesity, heart failure/ischaemic heart disease, and the presence of a wound (Table 6).

**Table 5.** Factors Associated with Chronic Edema (Univariate Analysis)

*Factors associated with the presence of CO*	*No edema*		*Chronic edema*		*OR (95% CI)*	p
	N	*%*	N	*%*		
Gender						
male	496	45.1	598	41.5	1.00	
female	604	54.9	842	58.5	1.15 (0.99–1.35)	0.072
Age (years)						
<30	24	2.2	10	0.7	1.00	
30–39	31	2.8	13	0.9	1.01 (0.38–2.69)	
40–49	59	5.4	48	3.3	1.95 (0.85–4.48)	
50–59	98	8.9	116	8.1	2.84 (1.30–6.23)	<0.001
60–69	150	13.7	186	12.9	2.98 (1.38–6.42)	
70–79	191	17.4	356	24.7	4.47 (2.10–9.55)	
80–89	379	34.5	522	36.3	3.31 (1.56–6.99)	
90+	166	15.1	189	13.1	2.73 (1.27–5.88)	
Ethnicity						
White	851	77.8	1258	87.4	1.00	
Asian	161	14.7	127	8.8	0.53 (0.42–0.68)	
Black	53	4.8	35	2.4	0.45 (0.29–0.69)	<0.001
Other	12	1.1	10	0.7	0.56 (0.24–1.31)	
Mixed	17	1.6	10	0.7	0.40 (0.18–0.57)	
Obesity						
Underweight	199	18.1	132	9.2	1.00	
Normal weight	752	68.4	805	55.9	1.61 (1.27–2.05)	
Obese	135	12.3	408	28.3	4.55 (3.39–6.11)	<0.001
Morbidly obese	13	1.2	95	6.6	11.01 (5.93–20.48)	
Lower limb mobility						
Walks unaided	418	38.1	409	28.4	1.00	
Walks with aid	440	40.1	760	52.8	1.77 (1.47–2.11)	
Chair bound	137	12.5	181	12.6	1.35 (1.04–1.75)	<0.001
Bed bound	103	9.4	89	6.2	0.88 (0.64–1.21)	

**Table 6.** Independent Risk Factors Associated with Chronic Edema (Multivariable Analysis)

*Independent risk factors*	*OR (95% CI)*	p
Site		
Site A	1.00	
Site B	1.54 (1.04–2.27)	0.024
Site C	1.35 (1.11–1.64)	
Age (years)		
<30	1.00	
30–39	1.14 (0.39–3.32)	
40–49	1.82 (0.73–4.54)	
50–59	2.58 (1.08–6.13)	<0.001
60–69	2.94 (1.26–6.86)	
70–79	5.24 (2.27–12.14)	
80–89	4.49 (1.90–10.31)	
90+	3.78 (1.62–8.85)	
Ethnicity		
White	1.00	
Asian	0.53 (0.40–0.71)	
Black	0.66 (0.41–1.07)	<0.001
Other	0.53 (0.19–1.48)	
Mixed	0.56 (0.23–1.36)	
Obesity		
Underweight	1.00	
Normal weight	1.64 (1.26–2.15)	
Obese	4.78 (3.45–6.64)	<0.001
Morbidly obese	10.34 (5.28–20.22)	
Heart failure/CHD		
Absent	1.00	
Present	1.90 (1.50–2.40)	<0.001
Wound		
Absent	1.00	
Present	4.49 (3.72–5.39)	<0.001

## Discussion

Results from this study support the first study hypothesis that the prevalence of CO in patients treated by community nurses is high and has a significant association with the population of patients with wounds. As predicted, there is a relationship with age that has been identified in other studies; however, in this population, the distribution of gender was different with more males affected than in other research within similar populations.^1,17^

Community nursing services are delivering care to some of the most unwell and frail patients within the NHS. The criterion for a home visit is that the patient is unable to reach a clinic and this is reflected in this study with >80% being seen at home. However, the results indicate that CO adds an additional burden that further reduces mobility compared with those who are unaffected, and that obesity and morbid obesity are more problematic in this patient population. This is a population with many coexisting conditions and risk factors that may play an important role in the mechanisms leading to CO.

The role of ethnicity has not been examined in previous studies of CO patients. In this study, the pattern of ethnicity did not reflect the ethnic distribution within the general population. The sample was largely white Caucasian with few patients from Africa or south Asia despite in Leicester >60% of the general population being from these ethnic minority groups. It is not possible to determine whether this indicates that these patients are not accessing community nursing care or whether ethnicity directly influences the prevalence of CO.

Within the primary LIMPRINT study, the acute hospital prevalence of Japan was very low (6%) compared with a prevalence in European populations.^18^ This may indicate the importance of factors such as obesity. However, in elderly care hospitals in Japan, the prevalence was >60%, indicating the link with increasing age and comorbidities.^19^ Previous research in leg ulceration found a similar population profile in an area of London with a high South Asian population.^20^ The role of ethnicity in CO requires further elucidation to understand its potential importance.

The study indicates that CO is not being effectively managed within primary care in the United Kingdom with patients requiring frequent nursing visits. The overall rate of cellulitis in this study (24.7%) is similar to that reported in the results from LIMPRINT (34%) study. Detection and correct classification of cellulitis are often difficult and will be influenced by the level of skill clinical staff hold locally in assessment and treatment. Cellulitis may be confused with a number of other conditions such as contact dermatitis, chronic lipodermatosclerosis, acute inflammatory episodes, and acute erythema from uncontrolled edema.^21^ The high prescription rate of repeat antibiotics indicates that this is an important clinical issue that must be addressed and is compounded by the high number of concurrent wounds.

The main limitation of this study is the inability to determine whether patients have a primary or secondary CO. Primary forms are due to developmental abnormalities in the lymphatic system, some of which are due to genetic causes.^22^ Secondary CO occurs due to damage to the lymphatic and venous system and is compounded by issues such as immobility and obesity. There is, however, increasing recognition that even in primary forms of lymphedema, lymphatic and venous abnormalities may coexist. The common pathway to the development of CO is complex and influenced by episodes of infection that further damage the lymphatic transport and the development of adipose tissue.

In addition, the use of polypharmacy is implicated in edema formation. Many drugs are associated with edema such as calcium channel blockers used for hypertension and corticosteroids used for inflammatory conditions. Cancer drugs such as the taxanes are known to increase edema as are neuroleptic medications.^23^ The true mechanisms that lead to CO in this heterogeneous population require further study. However, based on our current understanding, the main treatment for all types of CO irrespective of the underlying etiology requires appropriate compression and this is influenced by factors such as limb shape, tissue changes, and overall function, which are profoundly altered in such patients.

The magnitude of the number of patients with CO in this study highlights the need for a rapid improvement in services that link acute and primary care. Initial studies in the United Kingdom indicate that integrated services with access to specialist advice can provide cost-effective outcomes and improved quality of life.^17^ The financial burden of managing patients with a wound was assessed during 2012–2013 and estimated at £2.2 million with 66% of the financial burden falling to community nursing services and general practitioners.^24^ The main cost of £1.94 million was attributed to leg ulcers (projected number 731,000 ulcers). When factoring the cost of comorbidities for this group, the cost increased to 5.3 billion or 4% of the public health expenditure at the time. This is similar in magnitude to the treatment of obesity. Despite this, 39% (0.9 million wounds) remained unhealed with the cost per patient ranging from £1719 to £5976. The costs drivers were related to nonhealing and the presence of diabetes and nutritional deficiency. Wound management is predominantly a nurse-led discipline but an estimated 30% of wounds lacked a correct diagnosis and compression was used in less than half. The study identified lack of access to specialist nurses as important issues affecting outcome and influencing costs. The results from this study support the magnitude of the problem and indicate that uncontrolled CO is an important factor contributing to poor wound healing. The use of prospective evaluation of all patients in this study rather than use of data from a primary care database wound indicate that the problem may be even greater than previously predicted.

## Conclusions

This study found that between 52% and 69% of patients cared for by community nurses have CO and of these 73% have a concurrent leg ulcer in the United Kingdom. Both clinical problems require effective compression therapy. Risk factor analysis has shown the importance of an increasingly aging and obese population with important comorbidities such as diabetes mellitus, heart failure, and reduced mobility. The projected changes in Western populations would indicate that CO is an important and expanding public health issue that must be urgently addressed.
